# Multi-Task Environmental Perception Methods for Autonomous Driving

**DOI:** 10.3390/s24175552

**Published:** 2024-08-28

**Authors:** Ri Liu, Shubin Yang, Wansha Tang, Jie Yuan, Qiqing Chan, Yunchuan Yang

**Affiliations:** School of Electrical and Information Engineering, Wuhan Institute of Technology, Wuhan 430205, China

**Keywords:** autonomous driving, environment perception, end to end, multi-task network

## Abstract

In autonomous driving, environmental perception technology often encounters challenges such as false positives, missed detections, and low accuracy, particularly in detecting small objects and complex scenarios. Existing algorithms frequently suffer from issues like feature redundancy, insufficient contextual interaction, and inadequate information fusion, making it difficult to perform multi-task detection and segmentation efficiently. To address these challenges, this paper proposes an end-to-end multi-task environmental perception model named YOLO-Mg, designed to simultaneously perform traffic object detection, lane line detection, and drivable area segmentation. First, a multi-stage gated aggregation network (MogaNet) is employed during the feature extraction process to enhance contextual interaction by improving diversity in the channel dimension, thereby compensating for the limitations of feed-forward neural networks in contextual understanding. Second, to further improve the model’s accuracy in detecting objects of various scales, a restructured weighted bidirectional feature pyramid network (BiFPN) is introduced, optimizing cross-level information fusion and enabling the model to handle object detection at different scales more accurately. Finally, the model is equipped with one detection head and two segmentation heads to achieve efficient multi-task environmental perception, ensuring the simultaneous execution of multiple tasks. The experimental results on the BDD100K dataset demonstrate that the model achieves a mean average precision (mAP50) of 81.4% in object detection, an Intersection over Union (IoU) of 28.9% in lane detection, and a mean Intersection over Union (mIoU) of 92.6% in drivable area segmentation. The tests conducted in real-world scenarios show that the model performs effectively, significantly enhancing environmental perception in autonomous driving and laying a solid foundation for safer and more reliable autonomous driving systems.

## 1. Introduction

In recent years, an increasing number of studies have shown that camera-based solutions for environment perception in autonomous driving have lower costs and provide efficient perception capabilities with the aid of deep learning. The main tasks of environment perception include traffic object detection, drivable area segmentation, and lane line segmentation. These functions ensure the safety and accuracy of the driving process.

Many methods have been developed to handle these tasks individually to achieve autonomous driving systems, with notable results in recent years. For instance, object detection algorithms such as Faster-RCNN [1], YOLOF [2], and DDQ [3]; lane line detection algorithms such as UFLDv2 [4], GANet [5], and BEV-LaneDet [6]; and drivable area segmentation algorithms such as PEBAL [7], DiGA [8], and CPL [9]. However, these methods generally achieve good results in single-task scenarios. In the research of autonomous driving technology, multi-task network architectures, which process multiple perception tasks in parallel, accelerate image processing and facilitate effective information sharing by using the same feature extraction network for different tasks. This results in more accurate detection and faster inference efficiency.

Recently, multi-task network models such as MultiNet [10], YOLOP [11], and HybridNet [12] have achieved significant results on the BDD100K dataset. However, they also have some limitations. For example, HybridNet, using anchor-based heads, has demonstrated excellent performance in detection tasks. Nevertheless, when designing loss functions or neck structures, these methods often focus on specific tasks; YOLOP, for instance, designs separate loss functions for each segmentation task, compromising the model’s generality and requiring substantial time to create and adjust the loss functions and their parameters. These methods also suffer from poor detection accuracy under low-light conditions and for small objects, and they involve high computational redundancy due to complex image features.

Existing algorithms often suffer from issues such as feature redundancy, insufficient contextual interaction, and inadequate information fusion, making it challenging to efficiently perform multi-task detection and segmentation simultaneously. To address these problems, and given that YOLOv8 [13] employs advanced network architecture and optimization techniques, this paper proposes an end-to-end multi-task environment perception model, YOLO-Mg, designed to simultaneously conduct traffic object detection, lane line detection, and drivable area segmentation.

The main contributions of this paper are as follows:

1. By introducing a multi-level gated aggregation network, perform multi-scale feature extraction using convolutions with different kernel sizes and dilation rates. This captures multi-level contextual interactions, dynamically excludes irrelevant interactions, and addresses the problem of feature redundancy by reallocating multi-level features. This approach reduces model computation while enhancing feature extraction capability.

2. The weighted bidirectional feature pyramid network (BiFPN) structure is reconfigured to incorporate finer-grained small-scale information and higher-level scale information. A feature enhancement module is added to address the lack of inter-layer information fusion and the problem of simultaneously detecting objects of different scales. Learnable weights are used to understand the importance of varying input features, repeatedly applying top-down and bottom-up multi-scale feature fusion, thereby improving the accuracy of small object detection tasks.

3. For the detection part, in addition to the detection head, two additional segmentation heads are designed: one for lane line segmentation and one for drivable area segmentation. A learnable weight is introduced to determine whether to perform feature fusion, achieving dynamic control of network behaviour to some extent.

## 2. Related Work

### 2.1. Detection of Traffic Scene Objects

Object detection in traffic scenarios is one of the core issues in visual environmental perception for intelligent vehicles and is a prerequisite for achieving autonomous driving. Deep learning-based object detection methods have demonstrated superior performance in complex environments. For instance, Deng Wei et al. [14] proposed a joint semantic approach based on the Faster R-CNN framework. Improving the network model enhanced the ability to detect small-scale objects and associate pedestrians with semantic attributes through spatial relationships, thereby improving multi-object pedestrian detection performance. In the context of object detection in complex scenarios, Liu Chao et al. [15] developed a monocular vision-based framework to identify the spatiotemporal distribution of vehicle loads. This framework detects vehicle occlusion, generates accurate occlusion region masks, and restores occluded images, effectively transforming occluded vehicle images into non-occluded ones to facilitate object detection in occluded scenarios. Wu et al. [16] proposed a nighttime vehicle detection algorithm based on image translation techniques. By adjusting the training set size to create translation models with varying degrees of fit, they selected the optimal model based on performance evaluation. The translated dataset was then used to train a YOLO-v5-based object detection model, improving annotation accuracy and precision in nighttime object detection. Xu et al. [17] proposed a method that integrates keypoint detection with multiple deep learning models, enhancing detection accuracy and stability in complex traffic environments. These approaches enrich the research content of object detection algorithms and provide strong support for achieving efficient and accurate object detection in more complex and dynamic traffic environments in the future.

### 2.2. Drivable Area Detection

For drivable area detection, the SNE algorithm [18] can infer the spatial information of the road surface from dense depth images. This method first uses SNE to process depth images, then extracts features using a convolutional neural network (CNN), and fuses these features with those extracted from the original RGB images using another CNN, ultimately outputting the drivable area segmentation map. To address the computational cost and speed issues in drivable area detection, Asgarian [19] transformed the recognition of drivable areas into a row selection task, achieving efficient drivable area detection by selecting specific rows to detect their boundaries. Another approach involves unsupervised methods [20], which leverage the fusion of image and LiDAR data to detect drivable areas. Unlike existing methods, this approach does not rely on lane markings and can be applied to drivable area detection in scenarios without clear lane markers. However, these methods require classifying every pixel in the image, resulting in high computational demands and long processing times. Therefore, optimizing the computational speed of these methods to meet real-time requirements is crucial in real-time applications.

### 2.3. Lane Line Detection

For lane detection, deep learning-based methods can be primarily divided into 2D lane detection and 3D lane detection. The LaneNet algorithm [21] segments lanes and outputs two classes: background and lane. Lane pixels are clustered into different lane instances using the mean-shift algorithm. After identifying which pixels belong to which lane, each lane instance is converted into parameter descriptions via H-Net. However, the network model is quite large. The RESA algorithm [22] introduced a dual upsampling module, where each block consists of a dual upsampling encoder with coarse-grained and fine-grained branches performing two upsampling steps. This process eventually restores the feature map, which is one-eighth the original size, back to its original dimensions, and the final feature map is used to predict the presence and probability distribution of each lane. The Ganet algorithm [5] generates features through an FPN structure, refining each lane line from high-level to low-level features. Each head obtains contextual information from lane prior features and performs classification and regression on the lane priors. However, the performance deteriorates significantly in scenarios involving lane changes.

### 2.4. Multi-Task Network

Multi-task learning (MTL) is a machine learning approach that involves simultaneously learning a fixed number of related tasks. Its goal is to enhance algorithm efficiency, prediction accuracy [23], and generalization ability [24] by learning shared representations across multiple objectives. In the domain of autonomous driving perception, Marvin et al. [10] proposed the MultiNet architecture, designed for the joint tasks of classification, detection, and semantic segmentation. This architecture employs a shared encoder and three separate decoders for each task, enabling end-to-end training using MTL algorithms. Building on this, Qian et al. developed the DLT-NET [25] network, which retains the encoder-decoder structure of MultiNet and introduces context tensors between decoders to facilitate information sharing across different tasks. Subsequently, Dong et al. [11] introduced the YOLOP network for panoramic driving perception, which concurrently performs traffic object detection, drivable area segmentation, and lane detection. They also proposed a novel training paradigm that meets real-time processing requirements but still faces challenges such as false detections, misdetections, and low robustness. Due to the high computational demands of vehicular edge computing (VEC), multi-task networks require efficient task scheduling strategies to ensure that all tasks are completed within specified latency and reliability constraints. Wu et al. [26]. proposed a Lyapunov optimization-guided deep reinforcement learning (DRL) approach that transforms long-term constraints into short-term, manageable optimization problems. Subsequently, the Soft Actor-Critic (SAC) algorithm in DRL was employed to learn the optimal resource allocation strategy. This method effectively enhances the URLLC performance in heterogeneous VEC systems and significantly optimizes the resource allocation strategy.

## 3. Design of Multi-Task Perception Methods

### 3.1. Backbone Network

**Figure 1:**
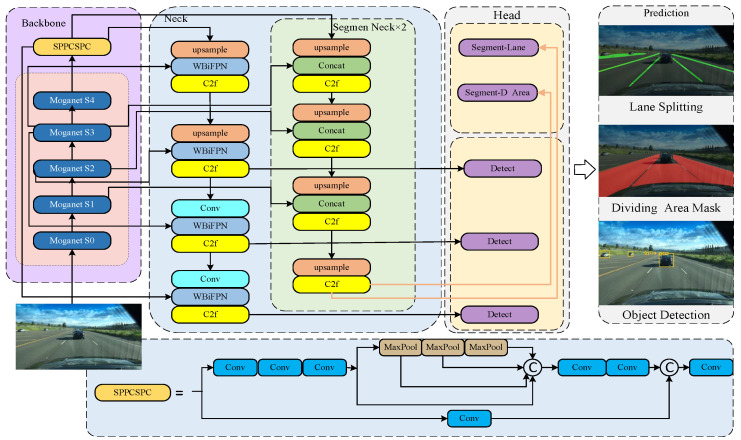
The architecture of the YOLO-Mg network.

**Figure 2:**
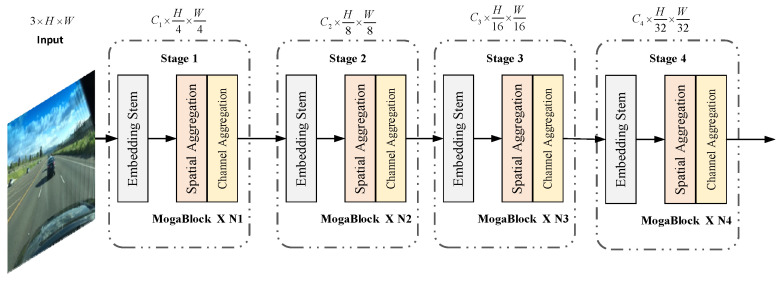
The architecture of the Multi-Order Gated Aggregation network.

The YOLO-Mg network structure is shown in Figure 1. It incorporates the Multi-Order Gated Aggregation (MOGA) network to reconstruct and enhance the weighted bidirectional feature pyramid network for feature fusion, and it is designed with three detection heads for detection. The backbone network can transform the input raw images into multi-layer feature maps for subsequent recognition tasks. This paper introduces a Multi-Order Gated Aggregation (MOGA) network [27] to replace the original backbone of YOLOv8. The structure of the MOGA network is illustrated in Figure 2.

The design of the MOGA network adopts a hierarchical approach, dividing the entire network into four consecutive stages. Each stage begins with an embedding module that transforms the input data into richer feature representations. Each stage includes several MOGA blocks that are responsible for processing and refining features. Each MOGA block comprises two primary modules: the spatial aggregation module and the channel aggregation module. The spatial aggregation module integrates information across spatial dimensions, aiding the network in understanding local structures and patterns within the image. Conversely, the channel aggregation module operates along the channel dimensions, redistributing and enhancing features to enable the network to capture the relationships among different features more effectively.

In the architecture of the Multi-Order Gated Aggregation (MOGA) network, the embedding module of the first stage consists of two 3 × 3 convolutional layers with a stride of 2. This setup performs preliminary feature extraction on the input feature maps while reducing their resolution. In the subsequent three stages, each embedding module similarly comprises two 3 × 3 convolutional layers with a stride of 2. Through processing these four stages, the resolution of the feature maps is progressively reduced to one-quarter, one-eighth, one-sixteenth, and one thirty-second of the original resolution.

**Figure 3:**
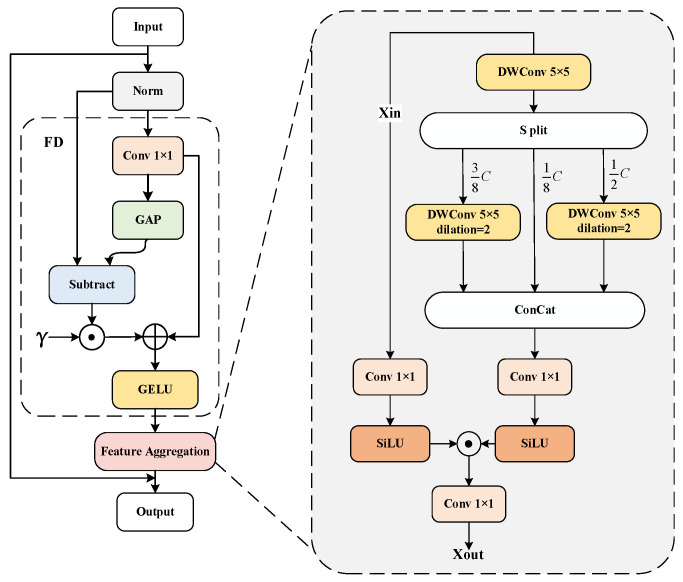
The spatial aggregation module.

The spatial aggregation module consists of the Feature Decomposition module (F.D.) and the Feature Aggregation module (F.A.), as illustrated in Figure 3.

The Feature Decomposition (F.D.) module is designed to capture both local details and the overall scope of input features, enabling the model to consider local textures and global information simultaneously. This process involves two main steps: first, a 1 × 1 convolution operation is applied to the input feature *X* to transform it into a new feature *Y*, as defined in Equation (1), thereby extracting local information; second, Global Average Pooling (GAP) is used to process the feature, capturing the overall statistical information, as defined in Equation (2) (where *γ* is a learnable parameter vector initialized to zero).

(1) $$Y=C{\mathrm{onv}}_{1\times 1}(X)$$

(2) FD(Y)=GELU(Y+γ⊙(Y−GAP(Y)))

The Feature Aggregation (F.A.) module is designed to capture and integrate contextual information at different scales, providing richer features to enhance model performance and accuracy. The module consists of a context branch and an aggregation branch. The context branch processes the input features (Xin) using three different sizes of depthwise convolutions. Initially, the input features are processed by a depth-wise convolution with a kernel size 5 × 5. The resulting features are then decomposed along the channel dimension into three parts: 1/8, 3/8, and 1/2, each retaining the original feature information.

Next, the 3/8 portion of the channels are input into a depthwise convolution with a kernel size of 5 × 5 and a dilation rate of 2. In comparison, the 1/2 portion is input into a depthwise convolution with a kernel size of 7 × 7 and a dilation rate of 3. These three parts of the features are then concatenated along the channel dimension and further processed by a 1 × 1 convolution in the aggregation branch to integrate them. Finally, the features from the aggregation branch are combined with the contextual information from the context branch to produce the output of the Feature Aggregation module, as illustrated in Equation (3). Here, *X* represents the output of the aggregation branch, *Y* represents the output of the context branch, and *Z* represents the output of the Feature Aggregation module.

(3) Z=SiLU(X)⊙SiLU(Y)

**Figure 4:**
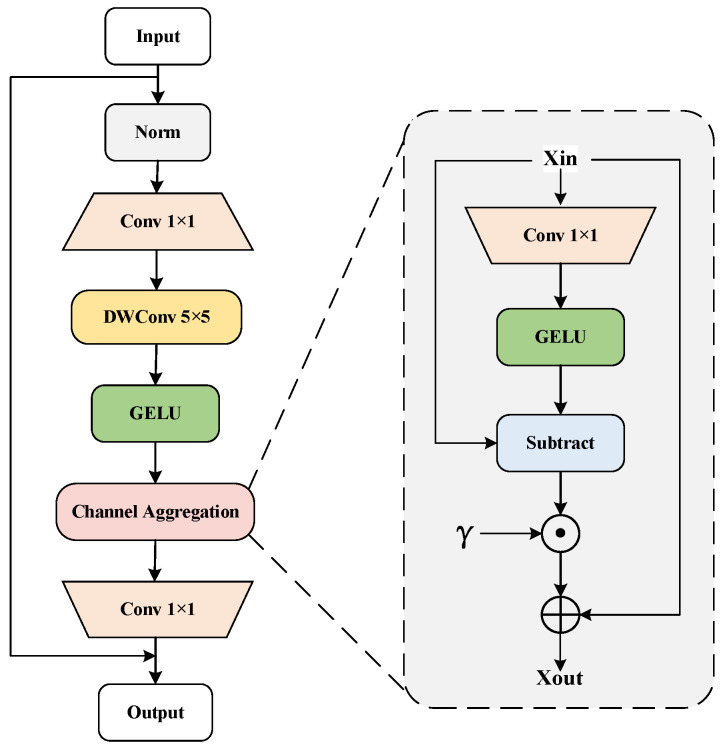
Channel aggregation module.

The structure of the Channel Aggregation (C.A.) module is depicted in Figure 4. This module comprises two layers of linear mixing with a specified channel expansion ratio. The channel information is aggregated by incorporating the Channel Aggregation module, enhancing the diversity of features along the channel dimension, as described in Equation (4).

(4) $$CA(X)=X+\gamma ⊙(X-GELU(Conv_{1\times 1}(X)))$$

Here, *X* represents the input features, and *γ* is a learnable parameter vector initialized to zero.

### 3.2. Feature Fusion Network

**Figure 5:**
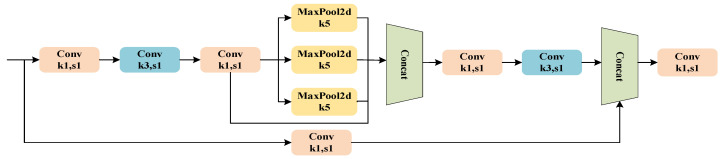
The structure of the SPPCSPC model.

**Figure 6:**
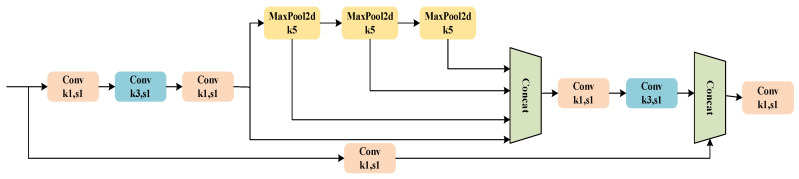
The structure of the reconstructed SPPCSPC model.

In the Feature Fusion Network, the role of the Spatial Pooling Module is to prevent feature information loss and to facilitate repeated feature extraction. Consequently, the SPPCSPC model, which is an improvement based on the SPPF module, is illustrated in Figure 5, and its reconstruction is shown in Figure 6.

The SPPCSPC consists of a Spatial Pyramid Pooling (SPP) component and a Cross Stage Partial Convolution (CSP) component. The CSP uniformly divides the input feature map into two parts. One part undergoes direct convolution processing to extract features, while the other part is processed through Spatial Pyramid Pooling (SPP). The SPP component employs pooling kernels of different scales to perform spatial pyramid pooling, which expands the model’s receptive field, allowing it to capture a wider region of the input data. Additionally, multi-scale pooling enhances the model’s feature representation capability, enabling it to understand better and process complex spatial structures.

As shown in Figure 6, the reconstructed Spatial Pooling Module replaces the original parallel 5 × 5, 9 × 9, and 13 × 13 max-pooling layers with three serial 5 × 5 max-pooling layers. Compared to the original structure, the reconstructed SPPCSPC module eliminates the need to perform different max-pooling operations on three separate threads, instead sequentially applying the same pooling operation three times. This change improves computational speed while maintaining the same level of accuracy.

**Figure 7:**
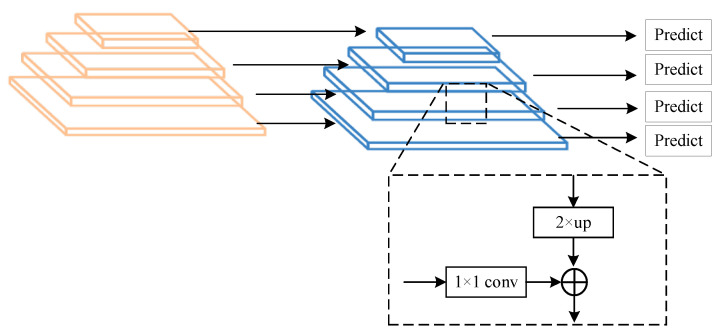
Schematic diagram of the FPN structure.

The Spatial Pooling Module transmits the processed feature information to the Feature Pyramid Network (FPN). The FPN uses a top-down strategy for feature information transfer. However, this unidirectional transfer prevents high-level feature information from being passed down to the lower feature layers, which may result in the loss of important information. The structural diagram is shown in Figure 7.

**Figure 8:**
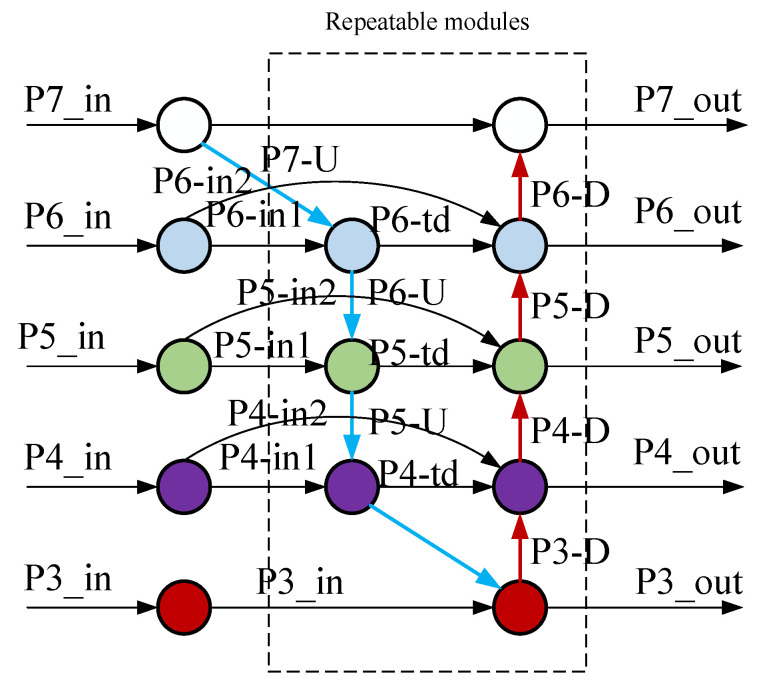
Diagram of the BiFPN structure.

The Bi-Directional Feature Pyramid Network (BiFPN) structure is improved based on the standard Feature Pyramid Network (FPN) architecture to achieve this. The standard Bi-Directional Feature Pyramid Network (BiFPN) structure is illustrated in Figure 8. The improved version adds a skip connection layer at the input and output nodes, integrating more features without increasing the parameters or computational complexity. Additionally, it combines top-down and bottom-up approaches to achieve a more comprehensive integration of multi-scale feature information.

Taking the P5 layer’s P5 node in the aforementioned structure diagram as an example, the intermediate feature inputs for P5 are P5_in and P6_U. P5_out is the output feature of the P5 layer in the bottom-up pathway. The outputs are P5_in, P5_td, and P5_out, respectively. The two feature fusion formulas are shown in Equations (3) and (4).

(5) $$P_{5}^{out}=Conv\left(\frac{w_{1}^{\prime }\cdot P_{5}^{in}+w_{2}^{\prime }\cdot P_{5}^{td}+w_{3}^{\prime }\cdot Resize(P_{5}^{out})}{w_{1}^{\prime }+w_{2}^{\prime }+w_{3}^{\prime }+\varepsilon }\right)$$

(6) $$P_{5}^{td}=Conv\left(\frac{w_{1}\cdot P_{5}^{in}+w_{2}^{\prime }\cdot Resize(P_{6}^{td})}{w_{1}+w_{2}+\varepsilon }\right)$$

Here, Conv represents convolution, and Resize represents sampling, while w and we are learnable weights. Due to the different resolutions exhibited by various features during cross-scale fusion and their varying impacts on the fused multi-scale feature maps, a learnable weight is added to each input feature to facilitate the distinction of the importance of each input feature and to understand the contribution of each feature to the output result. The weighted feature algorithm is expressed in Equation (5).

(7) $$O=\sum_{i}\frac{w_{i}}{\sum_{j}\varepsilon +w_{j}}\cdot I_{i}$$

**Figure 9:**
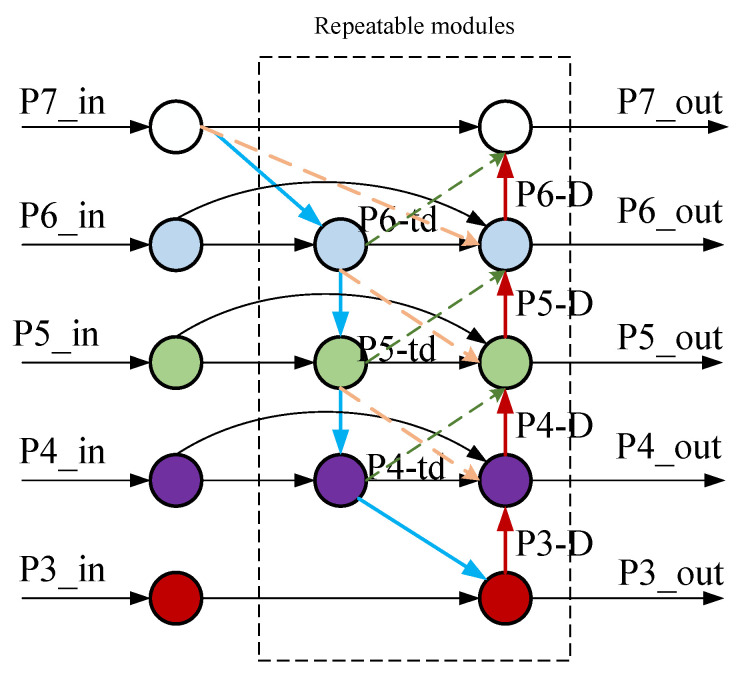
Reconstructed BiFPN structure diagram.

Here, *W_i_* and *W_j_* represent learnable weights. To avoid numerical instability, the learning rate ε is set to 0.0001. The index i denotes the input feature map at the *i*-th layer. Based on this, further reconstruction is performed, and the resulting structure is shown in Figure 9. 

The backbone network provides four different scales of features. In the BiFPN, each of the first *n* repeated modules has four inputs and outputs. P4 undergoes two repeated downsampling operations to obtain P5 and P6. The top-down feature fusion process is as follows:

(8) $$P_{4}^{td}=Conv\left(\frac{w_{1}\cdot P_{4}^{in}+w_{2}^{\prime }\cdot Resize(P_{5}^{td})}{w_{1}+w_{2}+\varepsilon }\right)$$

(9) $$P_{5}^{td}=Conv\left(\frac{w_{1}\cdot P_{5}^{in}+w_{2}^{\prime }\cdot Resize(P_{6}^{td})}{w_{1}+w_{2}+\varepsilon }\right)$$

(10) $$P_{6}^{td}=Conv\left(\frac{w_{1}\cdot P_{6}^{in}+w_{2}^{\prime }\cdot Resize(P_{7}^{td})}{w_{1}+w_{2}+\varepsilon }\right)$$

In the bottom-up pathway, the intermediate information at different scales is fully utilized for rapid normalized feature fusion, resulting in a single output from the reconstructed BiFPN. Taking P5 as an example, the output result is given by the following Equation:

(11) $$P_{5}^{out}=Conv\left(\frac{w_{1}^{\prime }\cdot P_{5}^{td}+w_{2}^{\prime }\cdot P_{6}+w_{3}^{\prime }\cdot Resize(P_{4}^{out})+w_{4}^{\prime }\cdot Resize(P_{5}^{out})+w_{5}^{\prime }\cdot Resize(P_{6}^{out})}{w_{1}^{\prime }+w_{2}^{\prime }+w_{3}^{\prime }+w_{4}^{\prime }+w_{5}^{\prime }+\varepsilon }\right)$$

By analogy, the outputs P3, P4t, P5t, P6, and P7 are obtained and used as inputs for the next layer. The reconstructed BiFPN integrates intermediate information from both lower and higher levels, fully utilizing multi-scale information to improve network performance, further enhancing the model’s capability for small object detection and its robustness.

### 3.3. Detection Network

The detection network will employ a detection head for scene object detection and two segmentation heads for lane line segmentation and drivable area segmentation, respectively. Additionally, an improvement to the neck involves introducing a learnable weight to determine whether feature fusion should occur, thereby achieving dynamic control over network behaviour to some extent. The detection process will adhere to the following principles:

1. During module initialization, the weight parameter W is set to 5.0, and then the weight is activated using the Sigmoid function. The activation function δ is defined as follows:
  (12) $$\sigma (w)=\frac{1}{1+e^{-w}}$$
2. Based on the activation result, the module path is selected as follows: if *δ(w)* > 0.5, a concatenation operation is performed; otherwise, the first input tensor is returned.
3. The input tensor list is concatenated along the specified dimension dim if concatenation is performed. The formula is as follows:
  (13) $$X_{concat}=Concat(x,dim)$$
4. Subsequently, the concatenated tensor *X_concat_* undergoes a convolutional layer operation. The formula is as follows:
  (14) $$Conv(x_{concat},\sum ch,ch[0],1,1)$$
  The term ∑*ch* represents the sum of the input channels, *ch*[0] indicates the number of output channels, and 1 denotes the size and stride of the convolution kernel.
5. Finally, the output is determined based on the result of the Sigmoid function, as expressed by the following formula:
  (15) $$Output=\left\{\begin{array}{cc} Conv(Concat(x,dim)) & \sigma (\omega )>0.5\\ x\left[0\right] & \sigma (\omega )\leq 0.5 \end{array}\right.$$

### 3.4. Loss Function

The loss function is composed of three parts: one for detection and two for segmentation. The specific formula is as follows:

(16) $$L=\alpha_{1}L_{\det}+\alpha_{2}L_{\mathrm{segda}}+\alpha_{3}L_{\mathrm{segll}}$$

$L_{\det}$ represents the object detection loss function, $L_{\mathrm{segda}}$ represents the drivable area segmentation loss function, and $L_{\mathrm{segll}}$ represents the lane line segmentation loss function. The object detection loss function is primarily composed of the binary cross-entropy loss $L_{\mathrm{bce}}$, which measures the classification error between the predicted and true labels, the distribution focal loss $L_{\mathrm{dfl}}$, and the complete IoU loss $L_{\mathrm{Iou}}$. Therefore, the formula for the object detection loss $L_{\det}$ is as follows:

(17) $$L_{\det}=\lambda_{\mathrm{bec}}L_{\det}+\lambda_{\mathrm{bfl}}L_{\mathrm{bfl}}+\lambda_{\mathrm{Iou}}L_{\mathrm{Iou}}$$

The loss coefficients are λ*_bce_*, λ*_bfl_*, and λ*_Iou_*. The formula for the binary cross-entropy loss $L_{\mathrm{bce}}$ is as follows:

(18) $$L_{\mathrm{bce}}=-\left[y_{n}\log x_{n}+(1-y_{n})\log(1-x_{n})\right]$$

The variable *X_n_* represents the predicted classification for each object, while *y_n_* denotes the actual label for each object. The distribution focal loss $L_{\mathrm{dfl}}$ measures the displacement between the predicted and true feature locations, ensuring that the predicted bounding box is closer to the actual bounding box. The formula is as follows:

(19) $$L_{dfl}=-(y_{i+1}-y_{i})log(\frac{y_{i+1}-y}{y_{i+1}-y_{i}})+(y-y_{i})log(\frac{y_{i}-y}{y_{i}-y_{i+1}})$$

Let y be a decimal representing the actual value of the bounding box coordinate and *y*_*i*+1_ and *y_i_* be its upper and lower bounds, respectively. The complete IoU loss, denoted as $L_{IoU}$, is formulated as follows:

(20) $$L_{IoU}=1-IoU-\frac{\rho^{2}(b,b^{gt})}{c^{2}}-\alpha \upsilon$$

(21) $$\alpha =\frac{\upsilon }{(1-IoU)+\upsilon }$$

(22) $$\upsilon =\frac{4}{\pi^{2}}{\left(arctan\frac{\omega^{gt}}{h^{gt}}-arctan\frac{\omega }{h}\right)}^{2}$$

Let b represent the predicted bounding box centre and *b^gt^* represent the ground truth bounding box centre. *p* denotes the Euclidean distance between the predicted and ground truth bounding box centres, and *c* represents the diagonal length of the smallest enclosing rectangle that covers both the predicted and ground truth bounding boxes. The parameters *α* and *ν* are used to control the scaling factors. *ω* and *h* denote the width and height of the predicted bounding box, respectively, while *w^gt^* and *h^gt^* denote the width and height of the ground truth bounding box. The loss function used for both drivable area segmentation and lane line segmentation is the same and is given by the following formula:

(23) $$L_{seg}=\lambda_{\mathrm{fl}}L_{\mathrm{fl}}+\lambda_{Tl}L_{Tl}$$

Here, $L_{\mathrm{fl}}$ and *Tl* represent the focal and Tversky losses, respectively.

(24) $$L_{\mathrm{fl}}=-\alpha_{t}{\left(1-p_{t}\right)}^{\gamma }log(p_{t})$$

Let *P_t_* represent the probability of the predicted positive class, *γ* be the base truth for each pixel, and α*_t_* be the weighting factor.

(25) $$L_{Tl}=1-\frac{TP}{TP+\alpha FN+\beta FP}$$

## 4. Experimental Validation and Results Analysis

This chapter will provide a comparative analysis of the datasets used in the experiments, the experimental parameters, and the model’s performance in scene object detection, drivable area segmentation, and lane line detection compared to other models. Additionally, it will present the results of ablation studies.

### 4.1. Experimental Setup

The experiments utilized image data from the BDD100K dataset to train and validate the model. The BDD100K dataset encompasses highly complex scenes with geographic, environmental, and weather diversity. It includes a substantial number of images of urban street scenes, covering various weather conditions, traffic situations at different times of the day, and a wide range of pedestrians and vehicles. Consequently, algorithms trained on this dataset exhibit significant robustness and can effectively transfer to new environments.

The recall rate (Recall) and mean average precision at an IoU threshold of 0.5 (mAP50) were used to evaluate the model’s performance as metrics for the object detection task. The mean Intersection over Union (mIoU) [28] was employed for the drivable area segmentation task, with higher values indicating better performance. For the lane line segmentation task, accuracy (Accuracy) and Intersection over Union (IoU) [29] were used as evaluation metrics, with higher values representing superior performance. The detection speed was measured in frames per second (FPS), indicating the number of images the object detection network can process per second; a higher FPS signifies faster image processing by the network model. The study employed the SGD optimizer with a learning rate set to 0.1, a batch size of 120, and 300 epochs. Experiments were conducted on a setup featuring a CPU configuration of 60 vCPUs (Intel(R) Xeon(R) Platinum 8352V CPU @ 2.10 GHz), Python version 3.10, Cuda version 11.8, and five RTX 4090 GPUs.

### 4.2. Experimental Results Analysis

#### 4.2.1. Object Detection

For the object detection task, the same settings as HyBridsNet and YOLOP were maintained to facilitate a better comparison. Specifically, I categorized cars, trucks, buses, and trains from the BDD100K dataset into a single ‘vehicles’ category. MAP50 and Recall were used as evaluation metrics for object detection. The results of the vehicle detection, compared with four models on the BDD100K dataset, are presented in Table 1.

As shown in Table 1, this model demonstrates superior detection performance compared to mainstream models, with a higher frame rate (FPS) than the two leading models. Specifically, the model achieves a 5% and 4.2% improvement in mAP50 over YOLOP and HybridNet, respectively. Additionally, it outperforms the more recent algorithms RegNetY and YOLO-ODL by 3.9% and 1.7% in mAP50, respectively.

#### 4.2.2. Lane Line Segmentation

To validate the model’s accuracy in lane line segmentation, it was compared with six algorithms: YOLOP, HybridNet, SCNN, Enet-SAD, RegNetY, and YOLO-ODL, as shown in Table 2. The model utilizes a restructured SPPCSPC module for feature extraction and an improved BiFPN for feature fusion, significantly enhancing the accuracy of small object detection. As indicated in Table 2, the model outperformed the leading algorithms, YOLOP and HybridNet, in accuracy and Intersection over Union (IoU). Specifically, the IoU increased by 3.3% compared to YOLOP and by 1% compared to HybridNet. Additionally, compared to the newer algorithms RegNetY and YOLO-ODL, the model showed improvements in accuracy by 2.7% and 4.6%, respectively, and an IoU increase of 1.4% compared to YOLO-ODL.

#### 4.2.3. Drivable Area Segmentation

In the drivable area segmentation section, the model was compared against seven algorithms: MultiNet, DLT-Net, PSPNet, YOLOP, HybridNet, RegNetY, and YOLO-ODL. The evaluation was conducted using the mean Intersection over Union (mIoU) metric, with the comparison results presented in Table 3. The network complexity was reduced by designing separate segmentation heads for drivable area and lane line segmentation. Since the difficulties of segmenting lane lines and drivable areas differ, independent segmentation methods can enhance the accuracy of each task. Additionally, the introduction of a learnable weighting mechanism aids in the dynamic fusion of features. As shown in Table 3, the model achieved mIoU improvements of 2.1% and 1.5% over HybridNet and YOLOP, respectively. Moreover, compared to the more recent algorithms RegNetY and YOLO-ODL, the model achieved mIoU gains of 0.5% and 0.3%, respectively.

### 4.3. Visualization Results

**Figure 10:**
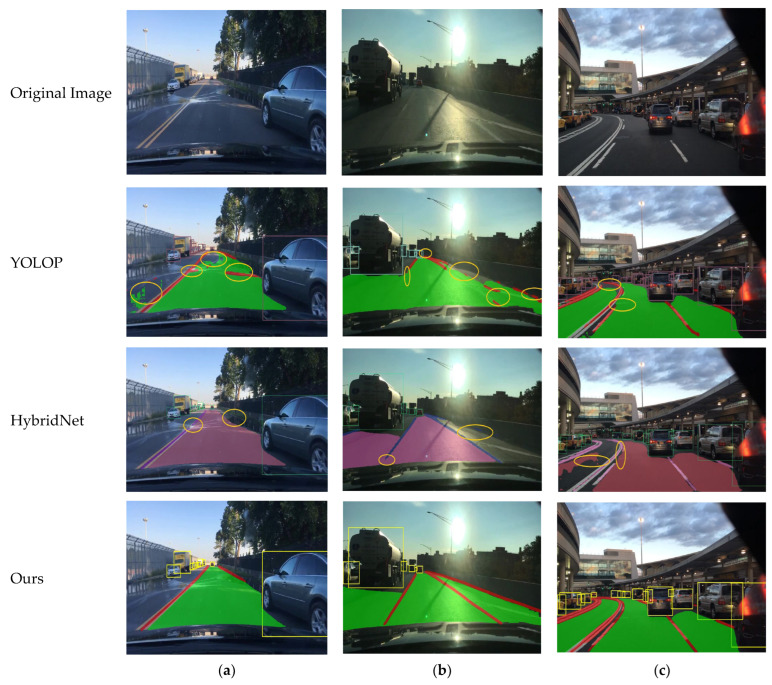
Visualization results in different daytime scenes: (**a**) represents typical daytime conditions, (**b**) represents daytime backlighting, and (**c**) represents daytime curves.

The visual comparison of the YOLOP, HybridNets, and YOLO-Mg models across various complex scenarios, as depicted in Figure 10, includes visual validation in three scenarios: normal daylight, backlit daylight, and curved daylight. The results demonstrate that in daylight conditions, YOLOP exhibits issues such as false positives on the left side, broken lane markings on the right side, and incomplete detection of drivable areas. In the backlit daylight scenario, due to image interference, YOLOP shows more pronounced lane marking breakage on the right side, with false positives occurring in the distant regions, and partial omission of drivable areas. In the curved daylight scenario, YOLOP fails to detect drivable areas on the left side in the distance due to the large curve radius. Despite good object detection performance, there are overlapping issues between lane detection and drivable area segmentation. For the HybridNets model, in the normal daylight scenario, lane markings are broken in high-light areas, and lane breakage is also observed in low-light areas; however, unlike YOLOP, no drivable area is missed. In the backlit daylight scenario, some drivable areas on the right side are undetected due to light interference, and lane markings in the near field are missing, although overall object detection performs well. In the curved daylight scenario, the left drivable area segmentation is similar to the backlit scenario, showing missing detections and suboptimal lane marking detection. The visual experiments conducted with the YOLO-Mg model in these three scenarios indicate that YOLO-Mg outperforms the other two algorithms in both backlit and curved scenarios, successfully addressing the issues they encountered.

**Figure 11:**
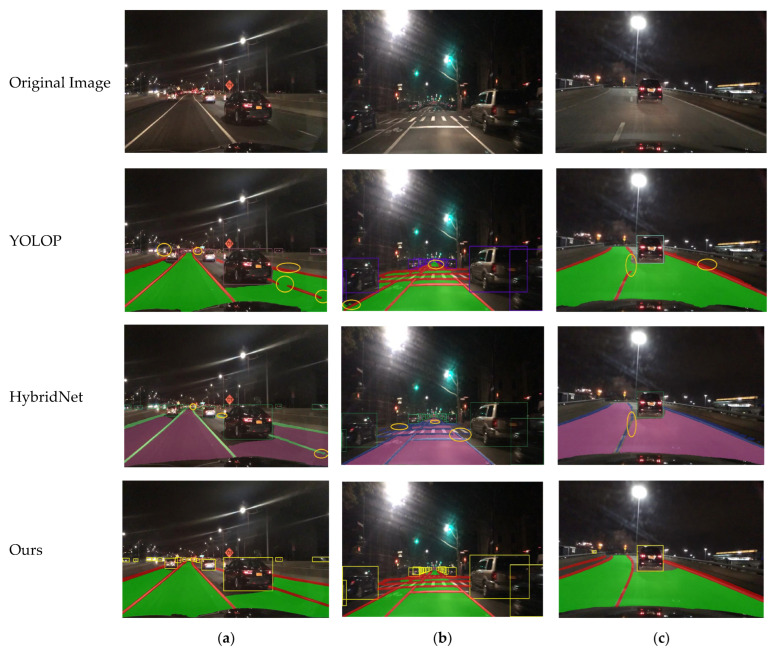
Visualization results in different nighttime scenes: (**a**) represents typical nighttime conditions, (**b**) represents backlit nighttime conditions, and (**c**) represents nighttime on a curved road.

As illustrated in Figure 11, we conducted visual verification of the algorithms in complex scenarios such as nighttime, backlit nighttime, and nighttime with curved roads. The analysis of the results reveals that the YOLOP algorithm exhibited lane line breakages on the left side even in clear nighttime conditions and failed to detect distant vehicle targets. In the backlit nighttime scenario, YOLOP demonstrated inaccurate detection of drivable areas, with lane line detection also suffering from breakages. In the nighttime curved road scenario, while YOLOP performed well in detecting drivable areas, it exhibited lane line breakages on the inner side of the curve. For the HybridNets algorithm, drivable area detection was effective in the nighttime scenario, but lane line detection showed breakages, and there were missed detections of distant vehicle targets. In the backlit nighttime scenario, lane line detection was not only inaccurate but there were also false detections in the drivable area. In the nighttime curved road scenario, lane line detection showed intermittent issues, though drivable area detection remained satisfactory. Visual experiments with the YOLO-Mg algorithm across these three scenarios indicate that, whether under backlit conditions or on curved roads, YOLO-Mg outperformed the other two algorithms and successfully addressed the issues they encountered.

**Figure 12:**
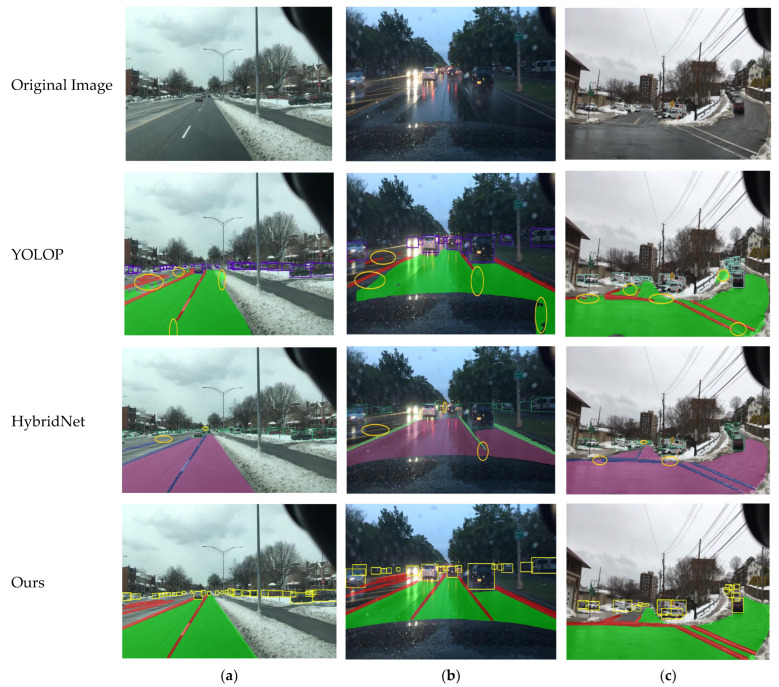
Visualization results in different rainy and snowy scenes: (**a**) represents snowy conditions, (**b**) represents rainy conditions, and (**c**) represents snowy curves.

As shown in Figure 12, we conducted visual tests of the algorithms in complex scenarios such as rainy weather, snowy weather, and snowy weather with curved roads. The results indicate that the YOLOP algorithm has certain shortcomings in snowy conditions, particularly in its failure to accurately detect the right-side lane line and its incorrect identification of the non-drivable area on the left as drivable. Additionally, the presence of interference factors affected the accuracy of object detection. In the rainy scenario, road reflections caused significant lane line breakages, although the detection of drivable areas and vehicle targets was relatively satisfactory. However, in the snowy curved road scenario, lane line breakages were again observed, and both object detection and drivable area recognition suffered from missed detections. In contrast, the HybridNets algorithm demonstrated better drivable area recognition in snowy conditions, though it also exhibited lane line breakages. In the rainy scenario, lane line detection still showed breakages, and the accuracy of detecting distant objects declined, although the recognition of drivable areas was acceptable. In the snowy curved road scenario, lane line breakages persisted, the detection of distant objects was inadequate, and false detections occurred in the drivable area. Visual analysis of the YOLO-Mg algorithm across these three scenarios revealed that, regardless of backlit or curved road conditions, YOLO-Mg outperformed the other two algorithms and successfully addressed the issues they encountered.

**Figure 13:**
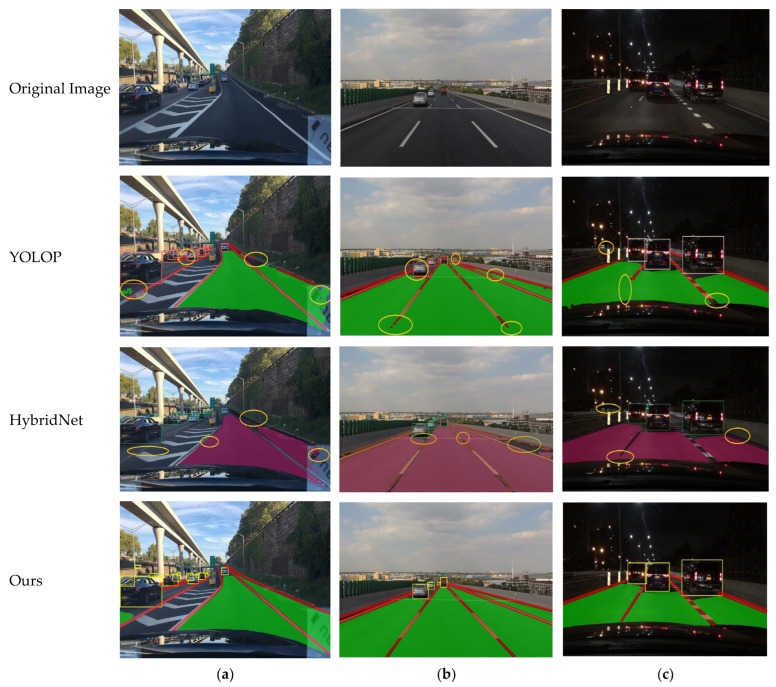
Visualization results in real-world scenarios: (**a**) represents a daytime junction, (**b**) represents daytime, and (**c**) represents nighttime.

In practical scenarios, we conducted visualized tests of the algorithms, covering test environments such as daytime intersections, daytime straight roads, and nighttime segments, as shown in Figure 13. The results indicate that the YOLOP algorithm exhibits certain deficiencies in daytime intersection scenarios, particularly with missed detections in the drivable area on the right side of the intersection, and the drivable area on the left side is not detected at all. Additionally, there are instances of lane line discontinuities, though object detection accuracy is not significantly affected. In the daytime straight road scenario, the algorithm shows instances of lane line discontinuities and partial missing of drivable areas. In the nighttime scenario, the algorithm exhibits lane line discontinuities and false detections in the left-side object detection. For the HyBridNet algorithm, in the daytime intersection scenario, there are issues with missing lane lines and undetected drivable areas on the right side, while the drivable area on the left side is completely undetected. In the daytime straight road scenario, there are missed detections in the drivable area on the right side, and the distant lane lines also show discontinuities. In the nighttime scenario, the left-side object detection exhibits false positives, but the drivable area detection performs well, although lane line discontinuities remain an issue. In contrast, the YOLO-MG algorithm effectively addresses these issues and demonstrates superior performance across various scenarios.

### 4.4. Ablation Study

The baseline network YOLOv8 has been improved in four aspects: replacing the backbone network with a Multi-Scale Gated Aggregation Network, modifying and reconstructing the feature fusion component SPPCSPC with a Weighted Bidirectional Feature Pyramid, and designing two additional segmentation heads. These improvements were sequentially incorporated into the baseline network, and the results obtained after training for the same number of epochs are presented in Table 4.

First, YOLOv8 was enhanced by adding two designed segmentation heads, creating baseline model A. The backbone network of model A was then replaced with a Multi-Scale Gated Aggregation Network (Moganet) to capture more context through multi-level interactions, resulting in model B. Compared to model A, model B exhibited a 5% increase in mAP50 and improvements of 1.3% and 1.2% in IoU and mIoU, respectively, indicating that the introduction of the Multi-Scale Gated Aggregation Network enhances the precision of feature extraction.

Next, the feature fusion module’s SPPF in model B was replaced with SPPCSPC, resulting in model C. Reconstructing the SPPCSPC module yielded model D. Comparing models B, C, and D reveals that while there was no significant change in accuracy, the reconstructed SPPCSPC module improved computational speed after three identical pooling operations.

Building on model D, a Weighted Bidirectional Feature Pyramid module was added, resulting in model E. A further reconstruction of the Weighted Bidirectional Feature Pyramid module produced model F. Comparing models D, E, and F shows that model E improved small object prediction capabilities, with a 4.2% increase in mAP50 and improvements of 3.8% and 2.9% in IoU and mIoU, respectively.

Finally, based on model A, the proposed model achieved a 9.6% increase in mAP50, a 4.8% increase in IoU, and a 4.6% increase in mIoU. This enhances the recognition of objects in driving environments for autonomous driving while the network achieves an FPS of 61, meeting certain real-time requirements.

## 5. Conclusions

This paper proposes the YOLO-Mg end-to-end network model to address the issues of inaccurate detection of small objects and the occurrence of missed and false detections in complex scenarios during environmental perception in autonomous driving. The network’s backbone is a multi-stage gated aggregation network, which employs convolutions with varying kernel sizes and dilation rates for multi-scale feature extraction. This design captures multi-level contextual interactions and dynamically filters out unimportant interactions. Redistributing multi-level features addresses the problem of feature redundancy often caused by traditional feed-forward networks (FFNs) in complex scenes. The feature fusion network is an improved weighted bidirectional feature pyramid network (BiFPN), which introduces finer-grained small-scale information and higher-level scale information, along with feature enhancement modules. This approach addresses the lack of inter-level information fusion and simultaneous detection of objects of different scales. The network learns the importance of varying input features through learnable weights. It repeatedly applies top-down and bottom-up multi-scale feature fusion, thereby improving the accuracy of small object detection tasks. Finally, the model includes one detection head and two segmentation heads for scene object detection, lane line segmentation, and drivable area segmentation. Experimental validation demonstrates that the proposed algorithm performs excellently across various scenarios, exhibiting a high degree of competitiveness. Future research will continue to investigate and empirically evaluate the performance of YOLO-Mg in a wider range of real-world driving scenarios. Additionally, efforts will be made to further optimize the network architecture with the aim of surpassing state-of-the-art (SOTA) algorithms and achieving a higher level of environmental perception. The detection capabilities under extreme conditions will also be further enhanced to advance the development of environmental perception technology for autonomous driving.

## Figures and Tables

**Table 1:** Comparison of object detection mAP50 and Recall data, with bold values indicating the best performance.

Model	mAP50	Recall	FPS
MultiNet	60.2	81.3	25
Faster R-CNN	64.9	81.2	19
YOLOV5s	77.2	86.8	**82**
YOLOP	76.4	88.6	51
HybridNet	77.2	89.9	29
RegNetY [30]	77.5%	86.1	-
YOLO-ODL [31]	79.7%	**94.2**	94
Ours	**81.4**	87.3	61

**Table 2:** Comparison of accuracy and IoU data for lane line segmentation, with bold values indicating the best performance.

Model	Accuracy	IoU
SCNN	35.78	15.48
Enet-SAD	N/A	16.02
YOLOP	76.4	25.6
HybridNet	77.2	27.9
RegNetY	76.9	**33.8**
YOLO-ODL	75.0	27.5
Ours	**79.6**	28.9

**Table 3:** Comparative mIoU data for drivable area segmentation, bold indicates the optimal values.

Model	Drivable mIoU
MultiNet	71.6
DLT-Net	72.3
PSPNet	89.6
YOLOP	91.1
HybridNet	90.5
RegNetY	91.9
YOLO-ODL	92.3
Ours	**92.6**

**Table 4:** Ablation study comparison, with bold values indicating the best performance.

Method	Model	FPS	mAP50	Lane IoU	Drivable mIoU
A	V8	83.7	71.8	24.1	88
B	V8 + Moganet	56.4	76.9	25.4	89.2
C	B + SPPCSPC	67.8	77.1	25.3	89.5
D	B + Improve SPPCSPC	67.1	77.2	25.1	89.7
E	D + BiFPN	61.9	80.8	28.5	90.8
F	D + Improve BiFPN	**61**	**81.4**	**28.9**	**92.6**

## Data Availability

No new data were created or analyzed in this study. Data sharing is not applicable to this article.

## References

[1] Ren S., He K., Girshick R., Sun J. (2016). Faster R-CNN: Towards real-time object detection with region proposal networks. IEEE Trans. Pattern Anal. Mach. Intell..

[2] Chen Q., Wang Y., Yang T., Zhang X., Cheng J., Sun J. You only look one-level feature. Proceedings of the IEEE/CVF Conference on Computer Vision and Pattern Recognition 2021.

[3] Zhang S., Wang X., Wang J., Pang J., Lyu C., Zhang W., Luo P., Chen K. Dense distinct query for end-to-end object detection. Proceedings of the IEEE/CVF Conference on Computer Vision and Pattern Recognition 2023.

[4] Qin Z., Zhang P., Li X. (2022). Ultra fast deep lane detection with hybrid anchor driven ordinal classification. IEEE Trans. Pattern Anal. Mach. Intell..

[5] Wang J., Ma Y., Huang S., Hui T., Wang F., Qian C., Zhang T. A keypoint-based global association network for lane detection. Proceedings of the IEEE/CVF Conference on Computer Vision and Pattern Recognition 2022.

[6] Wang R., Qin J., Li K., Li Y., Cao D., Xu J. (2022). BEV-LaneDet: A simple and effective 3D lane detection baseline. arXiv.

[7] Tian Y., Liu Y., Pang G., Liu F., Chen Y., Carneiro G. (2022). Pixel-wise energy-biased abstention learning for anomaly segmentation on complex urban driving scenes. Proceedings of the European Conference on Computer Vision.

[8] Shen F., Gurram A., Liu Z., Wang H., Knoll A. DiGA: Distil to generalize and then adapt for domain adaptive semantic segmentation. Proceedings of the IEEE/CVF Conference on Computer Vision and Pattern Recognition 2023.

[9] Wang Z., Zhao Z., Xing X., Xu D., Kong X., Zhou L. Conflict-based cross-view consistency for semi-supervised semantic segmentation. Proceedings of the IEEE/CVF Conference on Computer Vision and Pattern Recognition 2023.

[10] Teichmann M., Weber M., Zoellner M., Cipolla R., Urtasun R. Multinet: Real-time joint semantic reasoning for autonomous driving. Proceedings of the 2018 IEEE Intelligent Vehicles Symposium (IV).

[11] Wu D., Liao M.-W., Zhang W.-T., Wang X.-G., Bai X., Cheng W.-Q., Liu W.-Y. (2022). Yolop: You only look once for panoptic driving perception. Mach. Intell. Res..

[12] Vu D., Ngo B., Phan H. (2022). Hybridnets: End-to-end perception network. arXiv.

[13] Jocher G., Chaurasia A., Qiu J. (2023). Ultralytics YOLO (Version 8.0.0). https://github.com/ultralytics/ultralytics.

[14] Deng W., Liu B. (2018). Deep Learning-Based Pedestrian Detection Combined with Semantics. Comput. Syst. Appl..

[15] Xu B., Liu X., Feng G., Liu C. (2024). A monocular-based framework for accurate identification of spatial-temporal distribution of vehicle wheel loads under occlusion scenarios. Eng. Appl. Artif. Intell..

[16] Wu Y., Wang T., Gu R., Liu C., Xu B. (2024). Nighttime vehicle detection algorithm based on image translation technology. J. Intell. Fuzzy Syst..

[17] Xu B., Liu C. (2024). Keypoint detection-based and multi-deep learning model integrated method for indentifying vehicle axle load spatial-temporal distribution. Adv. Eng. Inform..

[18] Fan R., Wang H., Cai P., Liu M. (2020). Sne-roadseg: Incorporating surface normal information into semantic segmentation for accurate freespace detection. Proceedings of the Computer Vision–ECCV 2020: 16th European Conference.

[19] Asgarian H., Amirkhani A., Shokouhi S.B. (2021). Fast drivable area detection for autonomous driving with deep learning. Proceedings of the 2021 5th International Conference on Pattern Recognition and Image Analysis (IPRIA).

[20] Liu Z., Yu S., Wang X., Zheng N. (2017). Detecting drivable area for self-driving cars: An unsupervised approach. arXiv.

[21] Wang Z., Ren W., Qiu Q. (2018). Lanenet: Real-time lane detection networks for autonomous driving. arXiv.

[22] Zheng T., Fang H., Zhang Y., Tang W., Yang Z., Liu H., Cai D. Resa: Recurrent feature-shift aggregator for lane detection. Proceedings of the AAAI Conference on Artificial Intelligence 2021.

[23] Baxter J. (2000). A model of inductive bias learning. J. Artif. Intell. Res..

[24] Zhang Y., Yang Q. (2022). A Survey on Multi-Task Learning. IEEE Trans. Knowl. Data Eng..

[25] Qian Y., John M., Yang M. (2020). DLT-Net: Joint Detection of Drivable Areas, Lane Lines, and Traffic Objects. IEEE Trans. Intell. Transp. Syst..

[26] Wu Q., Wang W., Fan P., Fan Q., Wang J., Letaief K.B. (2024). Urllc-awared resource allocation for heterogeneous vehicular edge computing. IEEE Trans. Veh. Technol..

[27] Li S., Wang Z., Liu Z., Tan C., Lin H., Wu D., Chen Z., Zheng J., Li S.Z. Moganet: Multi-order gated aggregation network. Proceedings of the Twelfth International Conference on Learning Representations.

[28] Garcia-Garcia A., Orts-Escolano S., Oprea S., Villena-Martinez V., Garcia-Rodriguez J. (2017). A review on deep learning techniques applied to semantic segmentation. arXiv.

[29] Hou Y., Ma Z., Liu C., Loy C.C. Learning lightweight lane detection cnns by self attention distillation. Proceedings of the IEEE/CVF International Conference on Computer Vision (ICCV).

[30] Miraliev S., Abdigapporov S., Kakani V., Kim H. (2023). Real-time memory efficient multi-task learning model for autonomous driving. IEEE Trans. Intell. Veh..

[31] Guo J., Wang J., Wang H., Xiao B., He Z., Li L. (2023). Research on road scene understanding of autonomous vehicles based on multi-task learning. Sensors.

